# An inducible and reversible system to regulate unsaturated fatty acid biosynthesis in *C. elegans*

**DOI:** 10.1093/g3journal/jkaf025

**Published:** 2025-03-18

**Authors:** Bernabe Battista, Bruno Hernandez-Cravero, Monica P Colaiácovo, Luisa Cochella, Andres Binolfi, Diego de Mendoza

**Affiliations:** Institute of Molecular and Cellular Biology of Rosario, National University of Rosario (IBR-CONICET-UNR) Ocampo y Esmeralda, Rosario 2000, Argentina; Institute of Molecular and Cellular Biology of Rosario, National University of Rosario (IBR-CONICET-UNR) Ocampo y Esmeralda, Rosario 2000, Argentina; Department of Genetics, Blavatnik Institute, Harvard Medical School, Boston, MA 02115, USA; Department of Molecular Biology and Genetics, Johns Hopkins University School of Medicine, Baltimore, MD 21205, USA; Institute of Molecular and Cellular Biology of Rosario, National University of Rosario (IBR-CONICET-UNR) Ocampo y Esmeralda, Rosario 2000, Argentina; Argentinian Platform of Structural Biology and Metabolomics (PLABEM), Ocampo y Esmeralda, Rosario 2000, Argentina; Institute of Molecular and Cellular Biology of Rosario, National University of Rosario (IBR-CONICET-UNR) Ocampo y Esmeralda, Rosario 2000, Argentina

**Keywords:** *C. elegans*, inducible depletion of UFA, AID system, cold stress, fertility, lipid storage, lipid droplets, solution-state NMR spectroscopy

## Abstract

Unsaturated fatty acids (UFAs) play crucial roles in various physiological and pathological processes. In animals, these lipids are synthesized from saturated fatty acids through the action of delta 9 (Δ9) desaturases. In *C. elegans*, three Δ9 desaturases are encoded by the genes *fat-5*, *fat-6*, and *fat-7*. The presence of multiple Δ9 desaturases has posed a significant challenge in developing a rapid and efficient approach to control UFA production in *C. elegans* and other model organisms. Utilizing the auxin-inducible degradation system, we specifically targeted the *C. elegansfat-7* gene, responsible for the major stearoyl-CoA desaturase (SCD), while deleting *fat-5* and *fat-6*. This design resulted in a strain that can be reversibly depleted of UFAs in the cells of interest. Conditional depletion in all somatic cells exhibited a pronounced auxin-dependent defect in UFA production. Using this system, we uncovered an essential requirement for de novo UFA production during the L1 and L2 stages. Moreover, our results support a direct connection between UFA levels, fat storage, and increased lipid turnover. This system will enable further studies exploring the cellular and physiological consequences of impairing UFA biosynthesis at different developmental stages or in specific tissues.

## Graphical Abstract

**Graphical Abstract:**
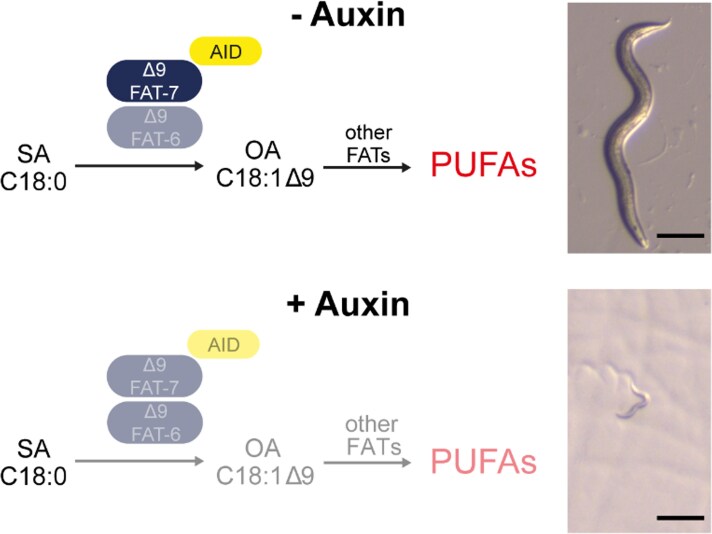

## Introduction

Lipids are key biologic molecules that contribute to a variety of cellular and organismal functions in 3 main manners (Watts and Ristow 2017). First, they are fundamental structural elements of cellular membranes, which build a selective barrier separating the cell from the environment and ensuring subcellular compartmentalization (Harayama and Riezman 2018). Second, they are key molecules in energy metabolism that fuel the cell (Watts and Ristow 2017; Yao *et al*. 2020). Third, they play active roles in signal transduction by either directly acting as signal molecules, or indirectly by affecting membrane fluidity (Kutchai *et al*. 1976; Kniazeva *et al*. 2004; Jeong *et al*. 2005; Zhu *et al*. 2013; Svensk *et al*. 2016; Busayavalasa *et al*. 2020). Thus, knowledge of the consequences of changes in lipid levels, composition and location has implications for understanding organismal biology in health and disease. A powerful animal model to address these issues is the nematode *Caenorhabditis elegans*.

**Fig. 1.**
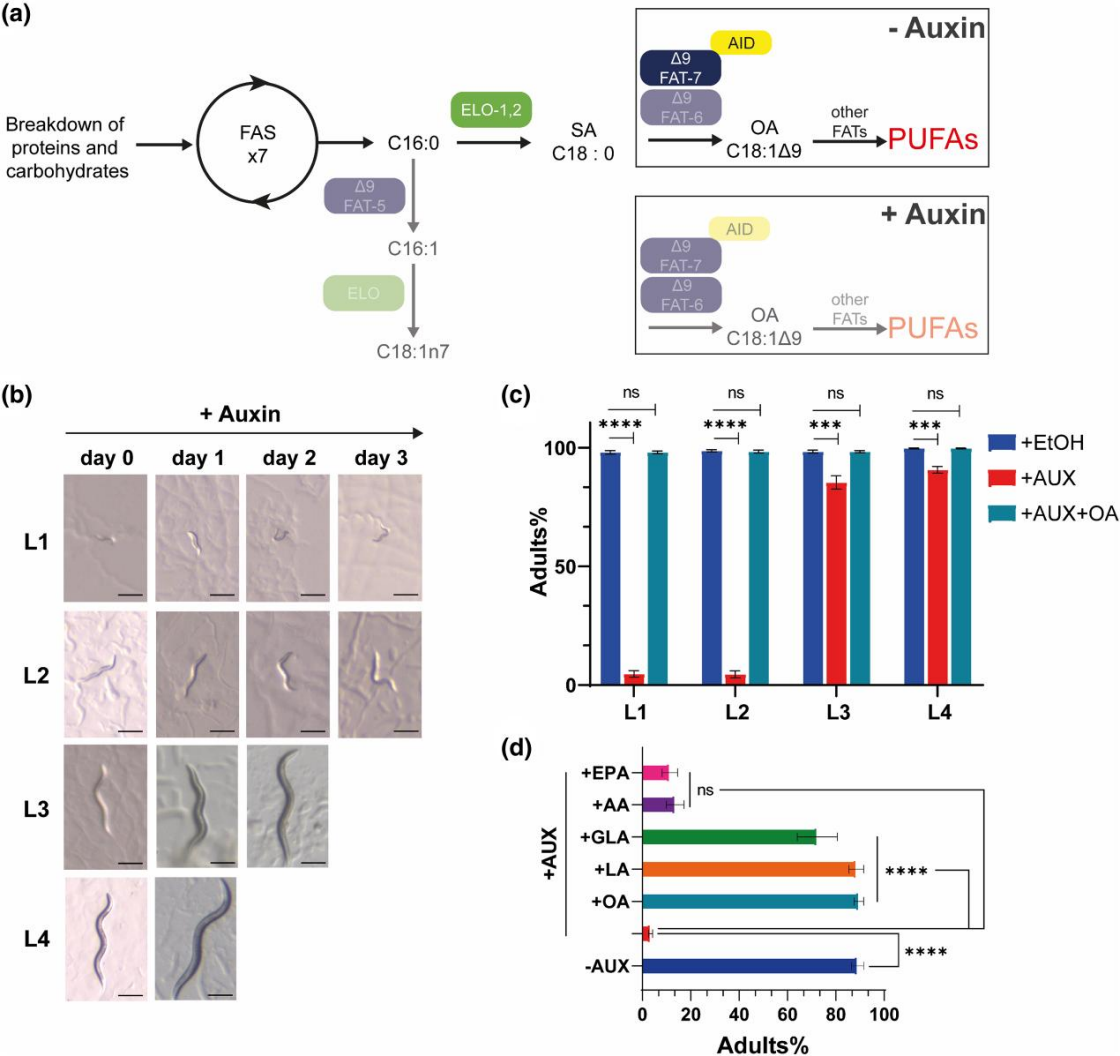
Auxin-induced interruption of UFA synthesis leads to developmental arrest in DDM6 worms. a) Simplified de novo fatty acid synthesis pathway of *C. elegans* DDM6 mutant strain in presence and absence of auxin. Enzyme names and activities are enclosed in ovals. FAS, fatty acid synthase; 16:0, palmitic acid, 16:1, palmitoleic acid, 18:1n7, *cis-*vaccenic acid; 18:1Δ9, oleic acid; PUFAs, polyunsaturated fatty acids. b) Representative images of L1, L2, L3, and L4 larvae growing on auxin-supplemented NGM plates for several days. Scale bar = 200 µm. c) Average percentage of adults reached by DDM6 L1, L2, L3, or L4 larvae incubated on auxin (AUX), ethanol, or auxin + fatty acid-supplemented plates at 20°C. Values significantly different from DDM6 + ethanol worms using unpaired t-test are (*) *P* < 0.05; (**) *P* < 0.01; (***) *P* < 0.001 and (****) *P* < 0.0001. Data shown are average of 3 independent experiments, each of 3 biological replicates; error bars indicate SEM. d) Average percentage of adults reached by DDM6 L1 larvae incubated on auxin with and without fatty acid supplementation. Values significantly different from DDM6 + auxin worms using non-parametric Kruskal–Wallis test followed by Dunn's multiple comparisons correction test, are (*) *P* < 0.05; (**) *P* < 0.01; (***) *P* < 0.001 and (****) *P* < 0.0001. Data shown are an average of 4–9 independent experiments, each of 3 biological replicates; error bars indicate SEM.

In practically all eukaryotic organisms, including *C. elegans*, unsaturated fatty acids (UFAs) are essential components of membrane and storage lipids. UFA synthesis depends on the conversion of saturated fatty acids by Δ9 desaturases (Brock *et al*. 2006, 2007). In *C. elegans* the *fat-6* and *fat-7* genes encodes a Δ9 stearoyl-CoA desaturase and a similar gene, *fat5,* encodes a Δ9 palmitoyl-CoA desaturase. The pathway for unsaturated fatty acid (UFA) synthesis in *C. elegans* begins with palmitic acid (16:0), obtained from the *E. coli* diet or synthesized de novo, which is converted to palmitoleic acid (16:1 Δ9) by FAT-5. This fatty acid is then elongated to cis-vaccenic (18:1Δ11), which is an abundant fatty acid in phospholipids and triglycerides. Palmitic acid (16:0) can also be elongated to stearic acid (SA, 18:0), the substrate for FAT-6 and FAT-7 desaturation to oleic acid (OA, 18:1Δ9) (Fig. 1 and Supplementary Fig. 1). Unlike most animals, *C. elegans* possesses a Δ12 fatty acid desaturase, which allows it to synthesize all polyunsaturated fatty acids (PUFAs) from OA (Supplementary Fig. 1) (Brock *et al*. 2007; Watts and Ristow 2017). These PUFAs play crucial roles in membrane function and signaling (Watts 2016). Previous genetic and phenotypic studies showed that the 3 Δ9 desaturases single mutants, *fat5*, *fat6*, and *fat7* display few differences from the wild type because they compensate for the loss of one isoform by regulated induction of the remaining Δ9 desaturase genes (Brock *et al*. 2006; Brock *et al*. 2007). However, the Δ9 desaturation activity is essential, and *fat6; fat7 fat5* triple mutants that lack this activity are unable to survive unless they are supplemented with an exogenous fatty acid mix composed of monounsaturated fatty acids (MUFAs) and polyunsaturated fatty acids (PUFAs) (Brock *et al*. 2006).

Given that UFAs play pivotal roles in membrane biology, efforts have been undertaken in *C. elegans* to disrupt the synthesis of these fatty acids, aiming to investigate the cellular and physiological consequences of compromised membrane homeostasis. A recently employed strategy for diminishing UFA synthesis involves utilizing *paqr-2*, *paqr-1* double mutants (Svensson *et al*. 2011; Busayavalasa *et al*. 2020). PAQR-2 and PAQR-1 exhibit partially redundant functions in promoting fatty acid desaturation and the integration of UFAs into phospholipids (Svensk *et al*. 2013; Svensk *et al*. 2016; Busayavalasa *et al*. 2020; Devkota, Henricsson, *et al*. 2021). However, recent studies have demonstrated that in addition, PAQR-2 amplifies sphingosine 1-P (S1P) production in response to membrane rigidification. S1P, a vital bioactive lipid, actively participates in diverse cellular and physiological processes, including binding to transcription factors that boost the transcription of *fat-5*, *fat-6*, and *fat-7* in *C. elegans* (Svensk *et al*. 2013; Vasiliauskaité-Brooks *et al*. 2017; Pilon 2021; Ruiz *et al*. 2022). Consequently, *paqr* mutants display high pleiotropy, potentially complicating the interpretation of the physiological consequences of UFA deprivation (Devkota, Kaper, *et al*. 2021). As a result, the field of membrane lipids has thus far lacked a genetically defined animal model to selectively obstruct UFA biosynthesis. Such a model would prove invaluable in advancing our comprehension of the molecular underpinnings of numerous processes affected by UFAs.

*C. elegans* lacks fat storage cells and stores neutral lipids, mainly triglycerides (TAG) and low amounts of cholesterol and cholesterol esters, in intestinal and hypodermal lipid droplets (LDs) (Mak 2012). LDs supply fatty acids to β−oxidation processes for energy production and serve as shuttles for lipids and proteins among different tissue and sub-cellular compartments (Pol *et al*. 2014). *C. elegans* strains with impaired Δ9 stearoyl-CoA desaturase have reduced adiposity and LD size (Shi *et al*. 2013). Moreover, recent work showed that *C. elegans* PUFAs stimulate LD fusion and growth (Wang *et al*. 2022), and that FAT-7 is directly involved in regulating organismal lipid storage and LD dynamics in an S-adenosyl-methionine and phosphatidylcholine-dependent manner (Han *et al*. 2024). Thus, a conditional mutant where UFA synthesis is perturbed at selected developmental stages represents a powerful tool to understand the role of UFAs in lipid storage biology and metabolic syndromes, such as obesity (Xie *et al*. 2022).

Here, we used the auxin-inducible degradation (AID) system in *C. elegans* (Zhang *et al*. 2015) to create a strain in which *fat-7* can be conditionally depleted. When combined with a double mutant of *fat-5* and *fat-6*, this setup allows for the targeted, acute depletion of OA in cells that express the TIR1 auxin-dependent, ubiquitin ligase. We show that expression of TIR1 in somatic cells enables auxin-dependent conditional depletion of MUFA synthesis at different larval stages. Our characterization of the conditional mutant reveals an essential role for de novo synthesis of OA in the transition from L1 and L2 larvae to the adult stage. We also demonstrate that auxin-mediated L1 larval arrest is caused by the decrease of OA (18:1Δ9) or C18 diunsaturated fatty acids, rather than by a reduction of C20 PUFAs. Moreover, depleting FAT-7 at the L3 stage interferes with lipid storage by decreasing the size and TAG levels in LDs, a phenotype that is efficiently rescued by exogenous supplementation of OA. Finally, we show that exposure to auxin at the L3 stage induces sterility as shown by a marked decrease in hermaphrodite self-progeny, which can be reversed upon OA media supplementation. Together, our results demonstrate that conditional depletion of tagged FAT-7 in the absence of redundant enzymatic activities provides a powerful new tool for the spatiotemporal regulation and analysis of UFA function in a metazoan model organism.

## Materials and methods

### Nematode maintenance and strains

Unless otherwise noted, worms were grown on nematode growth media plates (NGM) at 20°C seeded with *E. coli*OP50 lawns as a source of food (Kenyon 1988). Bacterial cultures were grown overnight on LB-broth (BD-Difco). Auxin solution was prepared in ethanol as carrier; then, it was added to seeded NGM plates to a final concentration of 4 mM along with hexane. Fatty acid methyl ester was prepared in hexane from commercial pure fatty acid (see below) and was used to supplement the auxin plates to a final concentration of 200 µM. Control plates were prepared by adding the same amounts of ethanol and hexane as in the auxin plates to seeded NGM plates. All plates were freshly prepared and vented for about 1.5–2 hours to allow full solvent evaporation, then stored at 4°C protected from light and used within a week. N2 Bristol was employed as wild-type strain. The other strain used in this research was DDM6 [ieSi57 II; *unc-119(ed3)* III; *fat-6(tm331)* IV; *fat-7(dme1[fat-7::3xflag::aid]) fat-5(tm420)* V].

### Construction of *fat-7*::3Xflag::AID and generation of DDM6 strain by CRISPR/Cas9 genome editing

To generate a C-terminal AID-tagged *fat-7*, CA1200 [ieSi57(*Peft-3*::TIR1::mRuby)] animals were subjected to Cas9-mediated genome engineering via RNP-microinjection following the Co-CRISPR protocol with modifications (Kim *et al*. 2014; Dickinson and Goldstein 2016).

The Alt-R CRISPR/Cas9 system (Integrated DNA Technologies) was employed, and animals were injected with a mixture containing 300 mM KCl, 20 mM HEPES, 4μg/μl recombinant Cas9 (from *S. aureus*, purified in-house), 500 ng/μl TracerRNA, 100 ng/μl crRNA against the locus of interest as well as 100–200 ng/μl dsDNA repair template encoding the desired modification with 50-bp flanking homology upstream and 300-bp downstream (due to the high AT content of the 3′ UTR). The injection mix also contained 2 ng/µl of a *myo-2p::mCherry* co-injection marker to identify successfully injected animals. Guide RNAs used and sequences inserted (in bold) were as follows:

The crRNA targeting sequence used was 5′- CTGCCGGCTGATAAACTCAT-3′

The sequence of the repair template and the locus post-edit, as verified by Sanger sequencing, was as follows:

GATACTGCAGCTGTTCTTGGTCTTGTCTACGATAGAAAAACAATTGC**t**GA**c**GA**a**TT**c**ATCAGCCGGCAGGTTGCCAATCACGGAAGTGAAGAATCAAGAAAAAAATCGATCATGGGAGGAAGCGGAGGAGGAAGCGGA**GACTACAAAGACCATGACGGTGATTATAAAGATCATGACATCGACTACAAGGATGACGATGACAAG*ATGCCTAAAGATCCAGCCAAACCTCCGGCCAAGGCACAAGTTGTGGGATGGCCACCGGTGAGATCATACCGGAAGAACGTGATGGTTTCCTGCCAAAAATCAAGCGGTGGCCCGGAGGCGGCGGCGTTCGTGAAG***TAAattcattatcattacgcgttggttgtccataaaagttttaattgaagcaaaaaataattaatttattcctgaataaatttggttaattctatgtataaacatcccactttaaaatttttgaaaaatcagtagaaaagtaaacgaaaaaagtttatttcaaagtttgtataaatcagacaatggttttgatatttttcataaaaacgaatatttcaatcataatgatgtaattttttgcaaaaagagttctgcttgttcttctacgttctaattttcgtttctgatacatacaactctgaaaacgagt.

Uppercase: coding sequence; except for synonymous mutations to avoid crRNA cutting of repaired product, which are lowercase and bold. Underlined, linker; Bold, Flag Tag; Bold and italicized, AID peptide; Lowercase: 3′ UTR. Genotyping was done by PCR with the following primers: Forward, TGGTTGGAAGCCATACGATACTTCTG and reverse, GATCACCAGAAAGGTACGCATGAGTAG.

Once *fat-7(dme1[fat-7::3xflag::aid])* was generated, it was crossed into strain BX110 carrying *fat-6(tm331)* IV; *fat-5(tm420)* V deletions to generate the UFA deficient conditional mutant strain DDM6: ieSi57 II; *unc-119(ed3)* III; *fat-6(tm331)* IV; *fat-7(dme1[fat-7::3xflag::aid]) fat-5(tm420)* V.

### Western blot of FAT-7::3xflag::AID

Given the low levels of FAT-7 expression, an additional step of immunoprecipitation was added prior to western blot analysis according to Clark *et al*. (2023) with modifications. Briefly, whole worm lysates were prepared from L4 s, exposed to auxin or ethanol from the L3 stage, by freezing and then sonicating the worms in lysis buffer [50 mM Tris–HCL pH 9.3, 50 mM NaCl, 5 mM EDTA, 1 mM PMSF, and 1 mM protease inhibitor cocktail (Roche)]. Then, the lysate was incubated with triton X-100 (2% v/v final concentration) at 4°C for 2 h by gently rocking to solubilize membrane proteins. After centrifugation at 13,000 rpm for 20 minutes, the supernatant was incubated with anti-FLAG affinity resin (Pierce) at 4°C overnight by gently rocking. Resin was washed 3 times with T-TBS, and FAT-7::AID was eluted with elution buffer (0.1 mM Glycine-HCl pH = 3.3). Proteins were resolved by SDS-PAGE and then transferred onto polyvinylidene difluoride membranes (GE, 0.2-μm pore size) in transfer buffer (25 mM Tris–HCl of pH 8.3, 192 mM glycine, and 20% (v/v) methanol). The primary antibodies were used at the following dilutions: rabbit α-FLAG (Sigma, 1:500), and mouse α-β-actin (1:10,000). The secondary antibodies were Horseradish peroxidase-conjugated from Biorad (1:3000). The proteins were visualized with GE ECL-Western Blotting substrate, and the relative levels of expression were determined by densitometric analysis of the bands (Auxin condition relative to ethanol condition) using FIJI-ImageJ software.

### Fatty acid extraction for GC–MS

For fatty acid analysis, c.a. 40000 N2 or DDM6 L3 larvae were transferred to auxin/auxin + OA/ethanol plates and allowed to grow for 48 h (1-day-old adults) or 72 h (2-day-old adults). Then, the adult worms were harvested and washed three times with M9 to remove any excess bacteria. Worms were centrifuged, excess buffer was removed, and the pellet was transferred to a glass tube. Lipids were extracted with 3 mL of chloroform:methanol 1:2 mixture (Blight-Dyer method). Dibutylhydroxytolueno (BHT) was added as an antioxidant at a final concentration of 0.005% v/v. The samples were incubated overnight at −20°C and centrifuged, and the supernatants were transferred to a new tube; 1 mL of 1 M KCl 0.2 M H_3_PO_4_ and 1 mL chloroform were added to the samples before vortex and centrifugation for 2 min at 2000g. Finally, the organic phase was transferred to a new tube and dried under N2(g) flow.

### Fatty acid methyl-ester preparations for GC–MS

For the derivatization of fatty acids previous to GC–MS analysis, 2 mL of 2.5% H_2_SO_4_ in methanol were added to the lipid samples, the tubes were capped and incubated at 80°C for 2 h. The fatty acid methyl esters were extracted with 3 mL of a 1:1 mixture of hexane and 5% v/v NaCl, dried under N2(g) flow, and finally resuspended in 500 µL of hexane.

### Gas chromatography coupled to mass spectrometry (GC-MS)

The methyl-ester analysis was performed in Shimadzu GC-2010 Plus equipment. The column used was a Supelco WAX-10 (Sigma Aldrich) 100% polyethyleneglycol. The helium flux was 1 ml/min, and the heating program was 180°C (0–32 min) and then an increasing gradient of 3°C/min from 180°C to 240°C. The split was 1/30, and the ionization voltage was 70 eV with an ionic range from 50 to 600 Da. The ion specters were registered as relative abundance in the function of mass/charge (m/z). Peak assignment was done using the mixture of standards of fatty acid methyl esters PUFA3 (Matreya).

### Sample preparation for NMR experiments of ^13^C-isotopically enriched worms

^13^C-isotopically enriched worms for in vivo and lipid extracts experiments were prepared based on previously reported routines (Hernández-Cravero *et al*. 2024). Briefly, 60,000 synchronized DDM6 L1 larvae/sample were placed on NGM plates seeded with 20× pellets of an overnight culture of *E. coli*OP50 grown in a M9 minimal medium supplemented with ^12^C-D-glucose (Sigma) and 1 g/L of ^14^N-ammonium chloride (Merck) as sole carbon and nitrogen sources, respectively. When the worms reached the L3 stage, they were transferred to auxin/auxin + OA/ethanol NGM plates seeded with 20× pellet of an overnight culture of *E. coli*OP50 grown in a M9 minimal medium supplemented with ^13^C-D-glucose (Cortecnec, France) and 1 g/L of ^14^N-ammonium chloride (Sigma-Aldrich) as sole carbon and nitrogen sources, respectively. OA was not isotopically enriched. In all cases, M9 media was also supplemented with 20 mg/L uracil (Merck) to compensate for OP50 auxotrophy under this minimal media. Animals were grown until 1-day adulthood, collected in a 15-mL falcon tube, centrifuged for 2 min at 2000g to remove media, and washed twice on M9 buffer to remove remaining bacteria. Supernatant was discarded, and M9 buffer was added to complete a volume of 700 μL supplemented with 10% D_2_O (99.9%, Sigma). Afterward, worms were loaded in a 5-mm NMR Shigemi tube (without applying the plunge) and decanted to the bottom by gentle spinning.

### Fatty acid extraction for in vitro NMR experiments

For fatty acid analysis of *C. elegans* extracts, nematodes were grown until 1-day adult stage, as for GC-MS. Then, nematodes were harvested and washed 3 times with M9 to remove any excess of bacteria, centrifugated, and pellets were frozen at −80°C. The pellets were thawed, resuspended in 1.3 mL of pure methanol, separated in 3 tubes, and sonicated on ice (Bioruptor sonifier, Diagenode). To prevent samples from heating, periods of 30 s at maximum power sonication were alternated with sonication-free lapses of 1 min during 10 min. After sonication, 2.6 ml of chloroform and 1.3 mL 0.5 M KCl/0.08 M H_3_PO_4_ were added to a final ratio of 1:2:1. The solutions were first subjected to sonication in an ultrasonic water bath for 15 minutes, followed by 2 minutes of vortex mixing. They were then centrifuged at 2,000 g for 10 minutes to facilitate phase separation. The lower, hydrophobic phase was carefully transferred into a clean glass tube, dried under a nitrogen stream, and re-dissolved in 500 μL of deuterated chloroform containing 0.005% BHT (Butylated hydroxytoluene) (Sigma) to prevent lipid oxidation. Finally, the solution was transferred into 5-mm NMR tubes (Norell, USA) using a glass pipette.

### NMR spectroscopy

NMR spectra were registered at 25 ^o^C on a 700-MHz Bruker Avance III spectrometer and a 5-mm triple resonance TXI probe (^1^H/D ^13^C/^15^N). 1D ^1^H Spectra of non-isotopically enriched lipid extracts were registered with a 30-degree flip angle hard pulse sequence (zg30), 8 K points, recycling delay of 3 s, 512 scans, and spectral width of 16 ppm. Processing was done by zerofilling to 32k points followed by a qsine window function multiplication, Fourier Transform, phase, and baseline correction. 1D ^1^H spectra of live worms and supernatant samples were acquired using a pulse sequence with excitation sculpting for water suppression (zgesgp) (Hwang and Shaka 1995), 8 K points, a recycling delay of 1 s, 128 scans and spectral width of 16 ppm. Processing was done by zerofilling to 32k points following by a qsine window function multiplication, Fourier Transform, phase, and baseline correction. 2D ^1^H-^13^C HSQC spectra of ^13^C-isotopically enriched lipid extracts were acquired with a phase-sensitive pulse sequence and gradient pulses (hsqcgpph). We used 1 K and 256 points in ^1^H and ^13^C, respectively, 4 scans, and 16 and 160 ppm spectral width for ^1^H and ^13^C, respectively. ^13^C decoupling was done with the GARP sequence. The spectra were processed with qsine window function multiplications, Fourier Transform, and phase and baseline correction in both dimensions. 2D ^1^H-^13^C HSQC spectra of live worms were acquired on ^13^C-isotopically enriched animals, with a sensitivity-enhanced pulse sequence (hsqcetgpsisp2.2). We used 2 K and 256 points in ^1^H and ^13^C, respectively, 4 scans and 16 and 160 ppm spectral width for ^1^H and ^13^C, respectively. Experimental time was 36 min. ^13^C decoupling was done with the GARP sequence. After NMR acquisitions, the worms were transferred to an Eppendorf tube, the supernatant was separated by gentle centrifugation (10 min at 1000 g) and measured again to asses leakage and excretion.

### Growth analysis

Worms were synchronized by hypochlorite treatment (Porta-de-la-Riva *et al*. 2012). Eggs were allowed to hatch overnight in the M9 buffer, and L1 larvae were placed onto seeded NGM regular plates, so they continue their development. Synchronous populations of L1, L2, L3 and L4 were collected from those plates and transferred to auxin/ethanol/auxin + FAME and incubated at 20°C. The number of adult animals was scored within the following 3 days.

### Fertility analysis

To analyze the progeny of individual worms, L4 (grown on auxin/ethanol/auxin + OA from the L3 stage as described before) were isolated and moved to auxin/ethanol/auxin + OA fresh plates. After reaching reproductive age, adults were moved daily to a fresh plate for 48 h. The adult worms were removed before the total live progeny was counted. For the reversibility assays, L4 hermaphrodites (grown on auxin from the L3 stage as previously described) were isolated and transferred to auxin plates for 24 hours, and then moved to fresh control plates (+ethanol) daily for 48 hours (condition +/−). In the +/+ condition, after the initial 24-hour exposure to auxin, worms were transferred to freshly prepared auxin plates daily for 48 hours. Adult worms were removed before counting the total number of live progeny.

### Survival at low temperatures

Synchronous populations of L3 were moved to auxin/ethanol/auxin + OA and incubated at 20°C, 15°C and 10°C for several days. The number of live, non-arrested worms was counted on each plate when the control population (+ethanol) reached adulthood. These values are expressed relative to the number of live non-arrested worms counted at 20°C (Brock *et al*. 2007).

### Nile red staining and quantification

Adult worms were fixed and stained with Nile red as described by Nhan *et al*. (2019). For both total lipid intensity and lipid droplet size quantification, worms were imaged using a Nikon Eclipse 800 equipped with an Andor Clara digital camera at 10× and 40× magnification, respectively. For total fluorescence intensity determination, the intensity was relativized to the whole worm area for each animal (Nhan *et al*. 2019). Total lipid droplet size determinations were made similarly as described in Shi *et al*. (2013). Briefly, for each worm photograph, an 18 × 18 µm square was placed arbitrarily over the mid-intestinal region, and within that square, each visible Nile Red-stained droplet was determine automatically using the ImageJ software. For each worm, the average lipid droplet size was determined. Statistical comparisons (Kruskal–Wallis and Dunn's multiple comparisons test) were performed using GraphPad Prism 8.

## Results

### Depletion of UFAs induces phenotypic defects in *C. elegans*

Since a *fat-6; fat-7fat-5* triple mutant is unable to survive (Brock *et al*. 2006), we exploited the *Arabidopsis* auxin-inducible degron (AID) tool kit system to engineer the *fat-7* locus using CRISPR/Cas9 to generate a strain for conditional depletion of FAT-7 (Supplementary Fig. 2). AID relies on the exogenous expression of the plant F-Box protein TIR1, which mediates the depletion of the degron-tagged targets upon exposure to auxin (Zhang *et al*. 2015; Ashley *et al*. 2021; Table 1). We tagged endogenous *fat-7* with the TIR1 ubiquitin ligase-recognition peptide and expressed TIR1 ubiquitously in all somatic cells. Then, we combined these components with available *fat-6* and *fat-5* null alleles to generate a UFA deficient conditional mutant, hereafter referred to as DDM6 (Fig. 1a). To test whether this strain produced the auxin-dependent conditional lethal phenotype expected for an animal lacking all 3 Δ9 desaturases, worms were transferred to NGM plates seeded with *E. coli*OP50 supplemented with auxin or ethanol (control plates) at various developmental stages, from L1 to L4 (Fig. 1b and c). Auxin treatment initiated at the L1 or L2 larval stages caused complete developmental arrest, in contrast to control animals, which developed into adulthood. However, animals treated with auxin starting at the L3 or L4 stages successfully matured into adulthood. These findings suggest that de novo UFA synthesis is critically required during the L1 and L2 stages, whereas later developmental stages may rely on fatty acids synthesized prior to FAT-7 depletion induced by auxin. Alternatively, these results could indicate incomplete depletion of FAT-7 during later stages of development. To test this, synchronized DDM6 L3 animals were exposed to auxin, and FAT-7 levels were analyzed by Western blot at the L4 stage. Control animals were not treated with auxin. Western blot analysis revealed a 60% reduction in FAT-7 levels in auxin-treated animals compared to controls (Supplementary Fig. 3). Based on these results, we hypothesize that the remaining FAT-7 protein levels are insufficient to support growth during the L1 and L2 stages, or alternatively, that auxin treatment has a stronger effect during earlier larval stages. However, as will be shown later, this substantial reduction in Δ9 fatty acid desaturation observed at the L4 stage significantly impairs UFA synthesis, resulting in distinct phenotypes associated with this deficiency.

**Table 1.** Fatty acid composition of wild-type and DDM6 mutants for the different conditions evaluated. Data are weight percentages (mean + SEM) of 4–6 independent determinations of total worm fatty acids measured by gas chromatography.

Fatty Acid	DDM6 + EtOH	DDM6 + Aux	DDM6 + AUX + OA	N2 + EtOH
14:0 n	1.9 ± 0.7	1.8 ± 0.7	0.9 ± 0.09	2.0 ± 0.5
16:0 n	6.1 ± 1.7	7.8 ± 1.1	5.3 ± 0.4	6.0 ± 1.4
18:0 n	18.0 ± 2.9	35.3 ± 3.7 *	21.1 ± 0.7	13.3 ± 2.5
Total saturated	26.1 ± 5.2	44.9 ± 5.2	27.4 ± 1.1	21.3 ± 4.1
16:1 cis	0.7 ± 0.3	0.9 ± 0.3	0.6 ± 0.03	1.4 ± 0.4
18:1Δ9	4.0 ± 0.2	1.0 ± 0.1 ***	9.2 ± 0.9 **	3.0 ± 0.6
18:1Δ11	10.0 ± 3.6	10.5 ± 1.7	9.5 ± 0.5	10.2 ± 2.3
Total MUFA	14.8 ± 3.9	12.3 ± 1.9	19.4 ± 1.4	14.7 ± 2.7
18:2 n6	11.6 ± 2.1	9.7 ± 2.5	12.7 ± 1.6	11.2 ± 1.1
20:3 n6	1.8 ± 0.3	1.4 ± 0.6	1.4 ± 0.02	2.8 ± 1.0
20:4 n6	1.8 ± 0.3	1.4 ± 0.6	1.4 ± 0.02	1.2 ± 0.2
20:4 n3	4.3 ± 0.2	2.4 ± 0.4 *	4.2 ± 0.4	5.0 ± 1.1
20:5 n3	13.5 ± 1.5	5.8 ± 0.2 **	12.4 ± 0.8	11.7 ± 2.2
Total PUFA	34.6 ± 3.2	21.5 ± 3.1 *	32.9 ± 3.5	32.0 ± 4.7
Total UFA	49 ± 6.7	33.7 ± 4.9 *	52.3 ± 2.1	46.8 ± 7.2
C15 iso	6.3 ± 1.2	5.1 ± 0.9	3.6 ± 0.3	8.2 ± 1.3
C17 iso	8.5 ± 1.6	9.0 ± 1.5	4.6 ± 0.6	14.5 ± 1.5 *
Total branched	14.8 ± 2.8	14.1 ± 1.8	8.2 ± 0.9	22.7 ± 2.8
17Δ	6.0 ± 3.5	4.5 ± 1.1	10.2 ± 1.3	3.3 ± 1.1
19Δ	3.5 ± 2.8	2.6 ± 0.3	1.7 ± 0.4	5.8 ± 3.0
Total cyclopropane	9.5 ± 2.7	7.1 ± 1.4	11.9 ± 1.3	9.1 ± 2.2

For each determination, L3 larvae were placed on auxin, ethanol, or auxin + oleic acid-supplemented NGM plates until they reached the 1-day-old adult stage. 17Δ, 9,19-methylenehexadecanoic acid, 19Δ, 11,12-methyleneoctadecanoic acid. Values determined to be significantly different from DDM6 + EtOH worms using an unpaired *t*-test are (*) *P* < 0.05; (**) *P* < 0.01; (***) *P* < 0.001; and (****) *P* < 0.0001.

### Dietary supplementation of fatty acids suppresses the arrest phenotype depending on the number of atoms and degree of unsaturation

To investigate the potential reversal of the arrest phenotype and its specificity, we performed a dietary supplementation of different UFA methyl esters in L1 larvae treated with auxin (Fig. 1d). Consistent with the expected biosynthesis block, the developmental arrest observed in L1 or L2 larvae exposed to auxin was completely reversed upon OA supplementation. Furthermore, the L1 arrest of DDM6 exposed to auxin was restored with the exogenous addition of linoleic acid (LA, 18:2-n3) (Fig. 1d), the product of OA desaturation by the FAT-2 (Δ12) desaturase (Supplementary Fig. 1). Partial rescue was also achieved with gamma-linoleic acid (GLA, 18:3n6), the desaturation product of LA by the FAT-3 (Δ5) desaturase (Supplementary Fig. 1). In contrast, supplementation with C20 fatty acids from either the n-6 or n-3 series, such as arachidonic acid (AA, 20:4n6) and eicosapentaenoic acid (EPA, 20:5n3) respectively, did not reverse the arrest of L1 larvae induced by auxin exposure. This indicates that these PUFAs are incapable of compensating for the deficiency of other UFAs synthesized by upstream desaturases (Supplementary Fig. 1) (Vrablik and Watts 2013).

### De novo UFA synthesis is required for growth at low temperatures and fertility

**Fig. 2.**
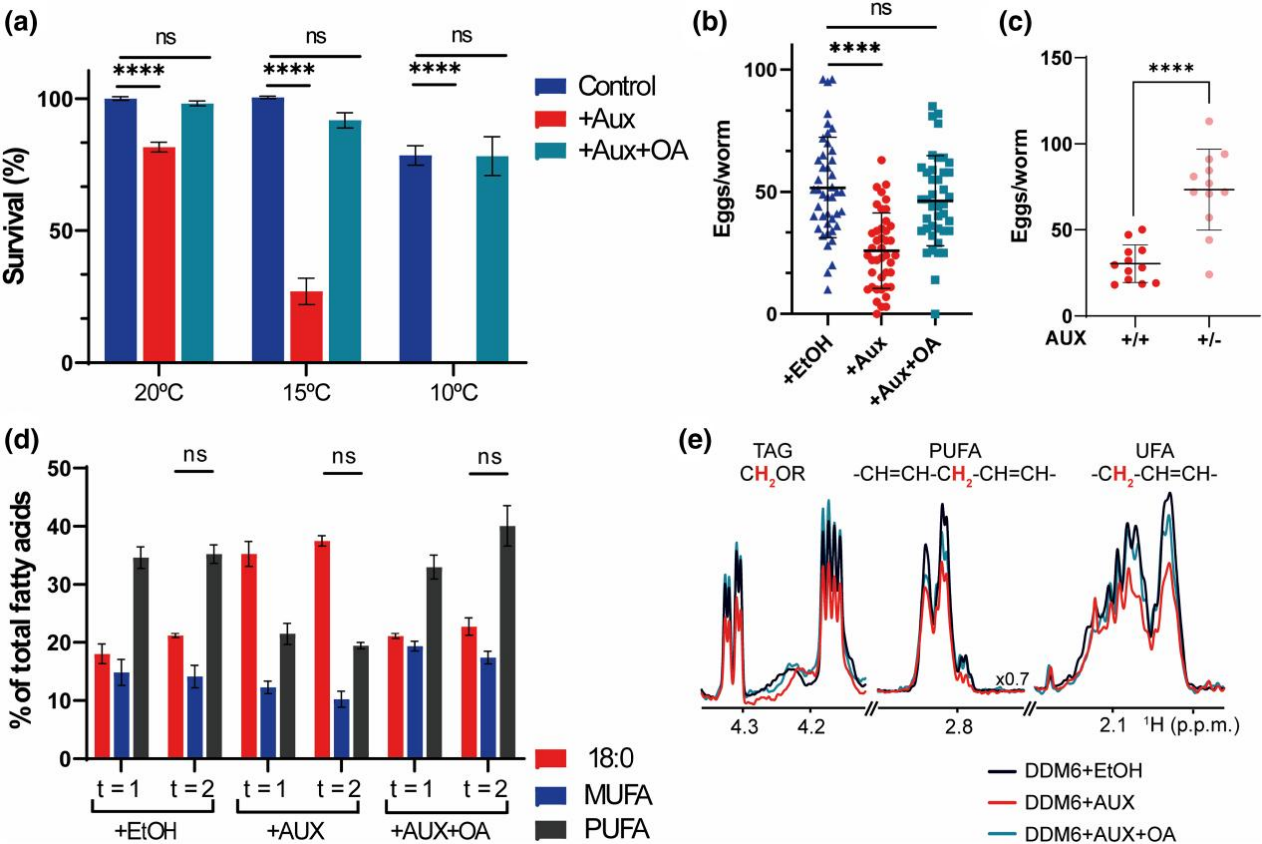
Reduced survival at low temperatures and fertility of DDM6 L3 larvae incubated with auxin. In all cases, DDM6 L3 larvae were placed on auxin, ethanol or auxin + oleic acid-supplemented NGM plates. a) Survival at 15°C and 10°C relative to that of 20°C worms. Values significantly different from DDM6 + ethanol worms using unpaired *t*-test are (*) *P* < 0.05; (**) *P* < 0.01; (***) *P* < 0.001, and (****) *P* < 0.0001. Data shown are an average of 2 independent experiments, each of 6 biological replicates; error bars indicate SEM. b) Average number of progeny produced by individual adults during a 48 h period. Values significantly different from DDM6 + ethanol worms using non-parametric Kruskal–Wallis test followed by Dunn's multiple comparisons correction test, are (*) *P* < 0.05; (**) *P* < 0.01; (***) *P* < 0.001, and (****) *P* < 0.0001, *n* = 40 individuals/condition, error bars indicate SD. c) Average number of progeny produced by individual adults during a 72-h period. In the +/+ condition, worms were exposed to auxin at all times, while in +/− condition worms were exposed to auxin for a 24-h window and then changed to control plates for 48 h hours. Values significantly different from +/+ worms using Welch's t-test are (****) *P* < 0.0001, *n* = 12 individuals/condition, error bars indicate SD (d) Simplified fatty acid composition of 1-day-old (t = 1) and 2-day-old (*t* = 2) worms. 18:0, stearic acid; MUFA, total monounsaturated fatty acids, PUFA, total polyunsaturated fatty acids. Values significantly different from 1-day-old worms of the same condition using unpaired *t*-test are (*) *P* < 0.05; (**) *P* < 0.01; (***) *P* < 0.001, and (****) *P* < 0.0001. Data shown are average of 4–5 independent determinations; error bars indicate SEM. e) 1D ^1^H NMR of *C. elegans* lipid extracts in the TAG, PUFA, and UFA spectral regions. The position of these regions in the spectra is indicated in Supplementary Fig. 3a (dotted squares). Extracts were prepared from non-isotopically enriched, DDM6 worms treated with ethanol, auxin, and auxin and oleic acid.

Previous studies have demonstrated that worms with impaired UFA synthesis exhibit sensitivity to cold stress (de Mendoza and Pilon 2019). To investigate whether reducing UFA synthesis in DDM6 plays a role in survival at low temperatures, L3 worms grown at 20°C were transferred to plates containing auxin, ethanol (control), or auxin-supplemented with OA and incubated at 3 different temperatures: 20°C, 15°C, and 10°C. We then scored the live, non-arrested worms after 2 days, which is the time it took control of L3 worms to reach adulthood. Setting the survival rate at 100% at 20°C for control worms, we observed 100 and 79% survival at 15°C and 10°C, respectively (Fig. 2a). However, worms placed on auxin plates showed a significant reduction in survival rates at low temperatures, with 81%, 27%, and 0% survival at 20°C, 15°C, and 10°C, respectively. In contrast, media supplementation with OA resulted in survival rates indistinguishable from those of the control group in all cases. Thus, interrupting de novo UFA biosynthesis at the L3 stage proves detrimental for nematodes at low temperatures.

Even at the permissive temperature of 20°C, we observed a reduced brood size in DDM6 worms upon FAT-7 depletion compared to the control (Fig. 2b). By counting the number of embryos produced by single worms during a 48-hour period, we found that DDM6 treated with auxin produced 50% of the progeny of the control condition (DDM6 + ethanol). Supplementation with OA restored the progeny rate to control levels in auxin-treated DDM6 worms (Fig. 2b), consistent with previous work showing that mutants impaired in PUFA synthesis have decreased lipid mobilization from the intestine to oocytes, affecting early oogenesis and reducing the brood size (Lynn *et al*. 2015; Devkota, Kaper, *et al*. 2021). We also found that the reduction in brood size induced by auxin is reversible. When hermaphrodites exposed to auxin for a 24-hour window were transferred to control plates (+ethanol), the number of embryos deposited on the media increased (Fig. 2c).

### Depletion of OA at the L3 stage alters fatty acid composition

To characterize the changes in lipid composition associated with the conditional depletion of OA, we transferred L3 larvae to auxin-containing plates until they reached the 1-day adult stage and then profiled fatty acids using GC/MS (Table 1). The DDM6 mutant, when supplemented with auxin, showed significant changes in fatty acid composition compared to the control worms, with a notable accumulation of stearic acid (SA, 18:0). SA comprised 35.3% of the total fatty acids, a marked increase compared to 18% in the control worms. Consistent with this, there was a marked reduction in OA levels and its derived PUFAs, such as eicosatetraenoic acid (ETA, 20:4n3) and EPA, following auxin-induced FAT-7 degradation. Supplementing with OA largely restored the fatty acid composition of auxin-treated worms to levels comparable to those of the control group (Table 1), confirming that auxin-mediated depletion of this MUFA alters PUFA production, which is responsible for the associated physiological defects. We found that longer incubation with auxin did not induce further alterations in the fatty acid composition (Fig. 2d, Supplementary Table 1). Thus, the SA, total MUFA, and total PUFA levels of 1-day-old adults exhibited only subtle differences compared to 2-day-old adult worms, when both were incubated with auxin starting at the L3 larval stage. The MUFAs and cyclopropane fatty acids (CFA) provided by the *E. coli* diet (palmitoleic acid, 16:1Δ9; cis-vaccenic acid, 18:1Δ11 and C17/C19 CFAs, Table 1) are insufficient to rescue the phenotypic defects, such as temperature sensitivity and reduced fertility in FAT-7 depleted worms (Figs. 2, a and b). This requirement for a specific level of C18:1Δ9, which cannot be met by the standard *E. coli* diet, was previously documented by Brock *et al*. (2016).

Independent ^1^H NMR experiments on extracts of unmodified lipids confirmed a decrease in the total contents of UFAs and PUFAs in DDM6 worms treated with auxin. We observed a reduction of ∼50% in the NMR signal amplitude of UFA and PUFA signals (Fig. 2e and Supplementary Fig. 4a) compared with control worms. Additionally, auxin treatment resulted in a similar reduction of triacylglycerol (TAG) molecules, as indicated by the lower intensity of the NMR signals corresponding to the -CH_2_OR- glycerol head group. The total levels of UFA, PUFA, and TAG were restored with the exogenous addition of oleic acid (OA), suggesting that FAT-7 activity is necessary for TAG biosynthesis and/or increased storage of neutral lipids.

### The interruption of UFA biosynthesis reduces adiposity in DDM6 worms

**Fig. 3.**
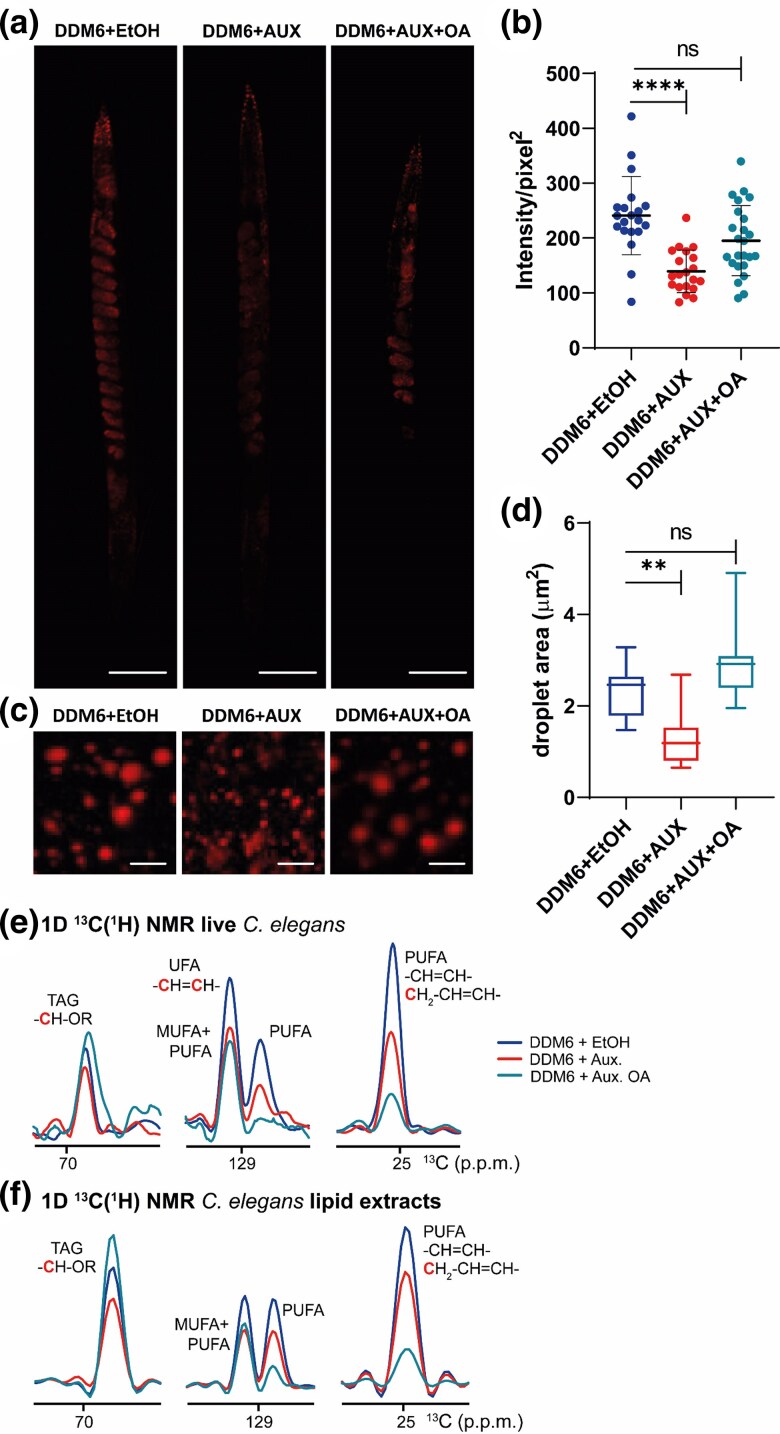
UFA synthesis interruption alters lipid storage. a) Fluorescent images of representative adult worms stained with Nile Red. Scale bars = 50 µm. b) Nile red staining quantification, each dot represents the mean intensity value corrected by area for every worm scored, *n* = 20, error bars represent SD. c) Fluorescent images of lipid droplets in the mid-intestinal region showing a range of droplets size. Scale bars = 5 µm. d) Quantification of lipid droplet size in the mid-intestinal region. For (b) and (d) values significantly different from DDM6 + ethanol worms using non-parametric Kruskal–Wallis test followed by Dunn's multiple comparisons correction test, are (*) *P* < 0.05; (**) *P* < 0.01; (***) *P* < 0.001, and (****) *P* < 0.0001. Data shown are an average of 2 independent experiments. (e, f) ^13^C NMR spectra of live worms (e) and total lipid extracts (f) in the TAG, UFA and PUFA regions. DDM6 worms were isotopically enriched with ^13^C and treated with ethanol, auxin, and auxin and non-isotopically enriched, ^12^C-oleic acid. ^13^C NMR traces were extracted from the 2D ^1^H-^13^C HSQC spectra shown in Supplementary Fig. 5.

We observed that DDM6 worms incubated with auxin often exhibit a clear intestine, a phenotype associated with reduced adiposity (Chen *et al*. 2019). To determine if this was the case, we used the lipophilic dye Nile Red to stain fat deposits and lipid droplets (LDs) in DDM6 animals whose UFA synthesis was interrupted at the L3 stage. Quantification of the total fluorescence confirmed that these worms had reduced fat levels compared to control worms, with about 60% of the total fluorescence intensity of the control group (Fig. 3, a and b). This feature was mainly evident in the embryos within the worm's uterus, that exhibited a fainter Nile Red staining in the auxin condition compared to the control. This suggests that the transfer of lipid resources from somatic cells to the germline, which is mediated by vitellogenins, is perturbed in the presence of auxin and requires precise UFA compositions (Chen *et al*. 2016).

In addition, DDM6 worms exposed to auxin had smaller LDs compared to control worms (Fig. 3c). The LD area in control worms ranged from 1.47 µm² to 3.28 µm², with an average size of 2.46 µm², whereas in auxin-treated DDM6 worms, the area ranged from 0.64 µm² to 2.68 µm², with an average size of 1.18 µm² (Fig. 3d). Dietary OA supplementation led to a partial recovery of total fat storage and restored lipid droplet size (Fig. 3, b and d).

We have recently demonstrated that multidimensional NMR spectroscopy, combined with uniform ^13^C-isotopic enrichment of *C. elegans*, enabled the dissection of lipid composition in LDs of live worms (Hernández-Cravero *et al*. 2024). Lipid analysis of live, ^13^C-isotopically enriched, auxin-treated animals confirmed a reduction in UFA, PUFA, and TAG (Fig. 3e and Supplementary Fig. 5, a and b), consistent with the decreased fat deposits and reduced LD size observed using fluorescence microscopy. NMR analysis of the supernatants confirmed that lipid signals originated from molecules within the intact worms (Supplementary Fig. 4b). Exogenous supplementation with non-isotopically labeled OA restored TAG levels but not UFA or PUFA levels, as described in more detail below. These observations were confirmed by complementary NMR experiments on total lipid extracts from ^13^C-isotopically enriched worms (Fig. 3f, and Supplementary Fig. 5, c and d). As mentioned above, upon OA supplementation of auxin-treated animals, TAG levels were restored but PUFAs were approximately 90% lower than those in ethanol-treated control animals and 40% lower than in auxin-treated animals without OA supplementation. This seemingly opposing behavior is explained by the fact that only endogenous TAG and fatty acyl chains that were generated during the growth on isotopically enriched bacteria are uniformly ^13^C-enriched and thus visible by NMR (Hernández-Cravero *et al*. 2024). Thus, the reduction in UFA and TAG levels observed in the presence of auxin directly reflects the decrease in these lipid species within the animals. In contrast, exogenously added OA is not isotopically enriched and thus remains NMR-invisible. As a result, newly synthesized lipid molecules that incorporate exogenous OA, such as UFA and PUFA fatty acyl chains, will not be detected in ^13^C correlation spectra (Supplementary Fig. 6). Therefore, the reduction in the PUFA signal following auxin/OA treatment, compared to auxin alone, suggests that the turnover of endogenous ^13^C-enriched PUFAs increases in the presence of OA, with new PUFAs being synthesized using exogenous OA as a substrate. This observation aligns with the decreased activity of FAT-7 in the presence of auxin.

## Discussion

In the present work, we have introduced a novel *C. elegans* strain that enables conditional depletion of UFA. Such strain, allows for a quick decrease of all sources of UFA, with a simple intervention: placing the worms on auxin-supplemented media at the desired larval stage. We successfully replicated the lethal phenotype of the *fat-6; fat-7fat-5* triple mutant (Brock *et al*. 2006) in DDM6 through auxin treatment, demonstrating the efficiency of depleting AID-tagged FAT-7. Moreover, we showed that the observed phenotypes upon auxin treatment were specific to the loss of Δ9 desaturase activity as they were rescued by supplementing the media with OA, the product of this enzyme. Moreover, GC-MS and NMR analysis on lipid extracts (Table 1 and Fig. 2e) revealed that the reduction in UFA content when worms reached the 1-day adult stage is due to a decrease in PUFA levels rather than MUFA content. We have also shown that longer exposure to auxin does not cause significant changes in the SFA/UFA ratio (Supplementary Table 1, Fig. 2d).

High-resolution in vivo NMR analysis of DDM6 worms treated with auxin confirmed the depletion of FAT-7 and a decrease in UFA and PUFA levels. Additionally, TAG content in LDs was lower in auxin-treated worms, further supporting the role of FAT-7 in regulating TAG biosynthesis and its incorporation into LDs (Shi *et al*. 2013; Wang *et al*. 2022; Han *et al*. 2024). OA supplementation restored TAG and UFA levels, as demonstrated by GC-MS and ^1^H NMR spectroscopy, and also replenished adiposity and LD content. Heteronuclear ^1^H-^13^C correlation NMR experiments revealed that the replenishment of fatty acyl chains in TAG molecules originated from exogenously added OA. Furthermore, OA addition resulted in a marked decrease of ^13^C-isotopically enriched PUFAs, which had been synthesized de novo by the worms, indicating an increase in lipid turnover stimulated by OA. These findings align with previous research showing that RNAi depletion of FAT-6 and FAT-7, which reduces OA and UFA production, significantly diminishes the incorporation rate of most fatty acids into membrane lipids (Dancy *et al*. 2015). Intriguingly, OA has been shown to accelerate β-oxidation of lipids through a Sirt-1/PGC1α-dependent mechanism in skeletal muscle cells (Lim *et al*. 2013), suggesting that the effects of OA on lipid turnover may be widespread among higher organisms. Our results support the use of NMR for monitoring lipid turnover (Lin *et al*. 2021, 2024) and highlight our system as an experimental model to further explore this mechanism, which remains poorly defined (Perez and Van Gilst 2008; Watts and Ristow 2017).

The depletion of UFA synthesis at the L3 stage showed noticeable phenotypes in DDM6 strain. These animals had dramatically reduced survival at low temperature (Fig. 2a) and reduced brood size (Fig. 2b). During oogenesis, the developing germ cells need a constant supply of resources to keep growing. To do so, somatic resources are transferred to the germline through the action of vitellogenins, which assemble and transport lipids as yolk from the intestine to the developing oocytes (Schneider 1996; Hall *et al*. 1999; Lynn *et al*. 2015). The transport of yolk to the oocyte relies on proper fatty acid composition (Chen *et al*. 2016), and conditional depletion of PUFA synthesis by auxin exposure could accumulate excess yolk material in the pseudocoelom leading to reduced brood sizes (Fig. 2b).

Previous work showed that a *fat-6; fat-7fat-5* triple mutant can grow when supplemented with OA, LA, and EPA (Brock *et al*. 2006). However, in that context, when L3/L4 larvae were transferred from supplemented to unsupplemented plates they became thin, sterile adults that died early (Brock *et al*. 2006). In contrast, DDM6 L3 larvae previously grown on control plates and then exposed to auxin remain viable and fertile, albeit with a smaller brood size, and their eggs develop into fertile adults on auxin-free plates (data not shown). This unique property of the AID system in DDM6 can be leveraged for future studies of germline defects induced by substantial reduction of de novo UFA biosynthesis.

Finally, we identified a critical role for endogenous OA (OA) production in facilitating the transition from the L2 larval stage to adulthood (Fig. 1, b and c). Interestingly, *fasn-1* RNA knockdown also leads to developmental arrest of *C. elegans* at the L2 stage (Li and Paik 2011). FASN is well known for its role in the biosynthesis of palmitate (16:0), a key building block of UFAs (Fig. 1a). However, a recent report demonstrated that beyond its essential role in the fatty acid synthesis, the C-terminal fragment of FASN-1 (FAS-CTF) also has a regulatory role in mitigating stress, which cannot be rescued by palmitate (Wei *et al*. 2024). Therefore, it is possible that, in addition to its structural role, oleate, or PUFAs—rather than stearoyl-CoA desaturase (SCD) alone—may have a regulatory function in the L2-to-L3 transition. However, the underlying mechanism remains unknown. One possible explanation is that OA or certain OA-derived molecules might act as intermediates capable of activating signaling pathways related to growth. In this context, previous studies have demonstrated the role of UFAs in modulating the activity of TRPV channels through sensory transduction (Kahn-Kirby *et al*. 2004) and the action of endocannabinoids (UFA-derived molecules) in regulating energy metabolism in *C. elegans* (Galles *et al*. 2018; Hernandez-Cravero *et al*. 2022).

In conclusion, we used the auxin-inducible degradation system targeting the *fat-7* gene in a double mutant (*fat-6*, *fat-5*) to develop a method that externally induces OA auxotrophy in *C. elegans*. We show that auxin exposure during larval development conditionally halts de novo UFA synthesis, which can be reversed upon cessation of auxin exposure or OA supplementation. This system is valuable for studying processes involving lipid storage, membrane remodeling (e.g. fluidity, curvature, vesicle and protein trafficking or signaling) and germline maintenance.

## Supplementary Material

jkaf025_Supplementary_Data

## Data Availability

Strains and plasmids are available upon request. The authors affirm that all data necessary for confirming the conclusions of the article are present within the article, figures, and tables. Supplemental material available at G3 online.
