# Supplementary material for: An inducible and reversible system to regulate unsaturated fatty acid biosynthesis in C. elegans
Source: G3 (Bethesda). 2025 Mar 18;15(4):jkaf025. doi: 10.1093/g3journal/jkaf025 (PMC12005147; doi:10.1093/g3journal/jkaf025)
Supplement: jkaf025_Supplementary_Data [file jkaf025_supplementary_data.zip › Supplemental_Material_G3-2025-405693.pdf]

Supplementary Data

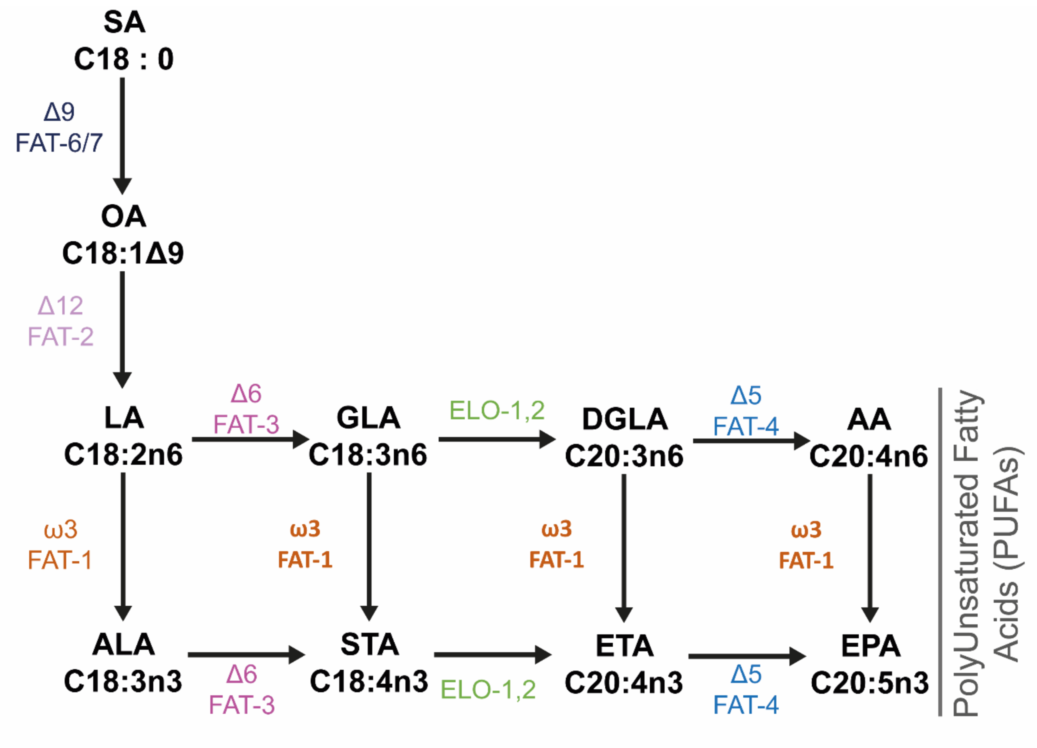

Figure S1. Simplified pathway of *de novo* fatty acid synthesis in *C. elegans*. Enzyme names and activities are depicted. ELO, elongase; SA, stearic acid; OA, oleic acid; LA, linoleic acid; ALA, alpha linoleic acid; GLA, gamma linoleic acid; STA, stearidonic acid; DGLA, dihommo gamma linoleic acid; ETA, eicosatetraenoic acid; AA, arachidonic acid; EPA, eicosapentaenoic acid.

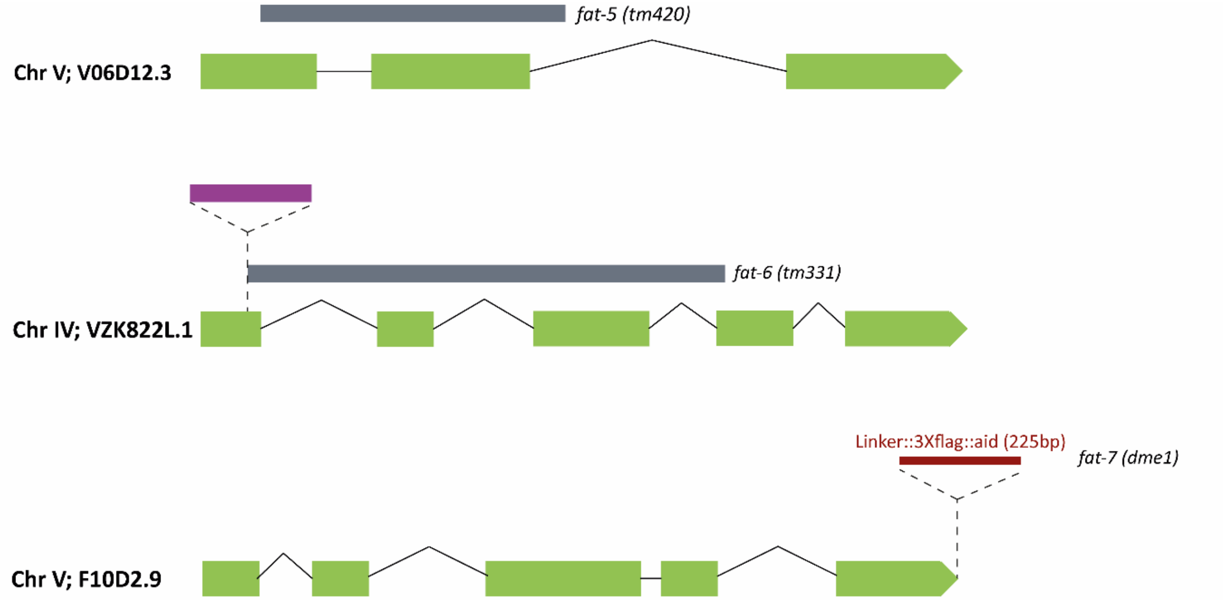

Figure S2. Diagram displaying Δ9-desaturases genes and mutations in DDM6 strain. The *fat-5(tm420)* mutation consists of a 779-bp deletion and *fat-6(tm331)* consist of a 1232-bp deletion (gray bar) plus a 429-bp insertion (purple bar) (Brock et al. 2006). The *fat-7(dme1)* allele consists of a 225-bp insertion (red bar) containing the 3XFLAG and AID recognition sequences.

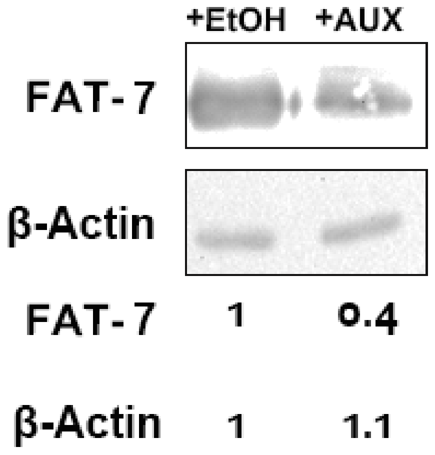

Figure S3. Western blot analysis of FAT-7::3xflag::AID in DDM6 worm lysates detected with an anti-FLAG antibody (FAT-7) and anti-β-actin antibody (β-Actin). The level of each protein relative to EtOH condition was determined by densitometric analysis of the western blot bands with ImageJ.

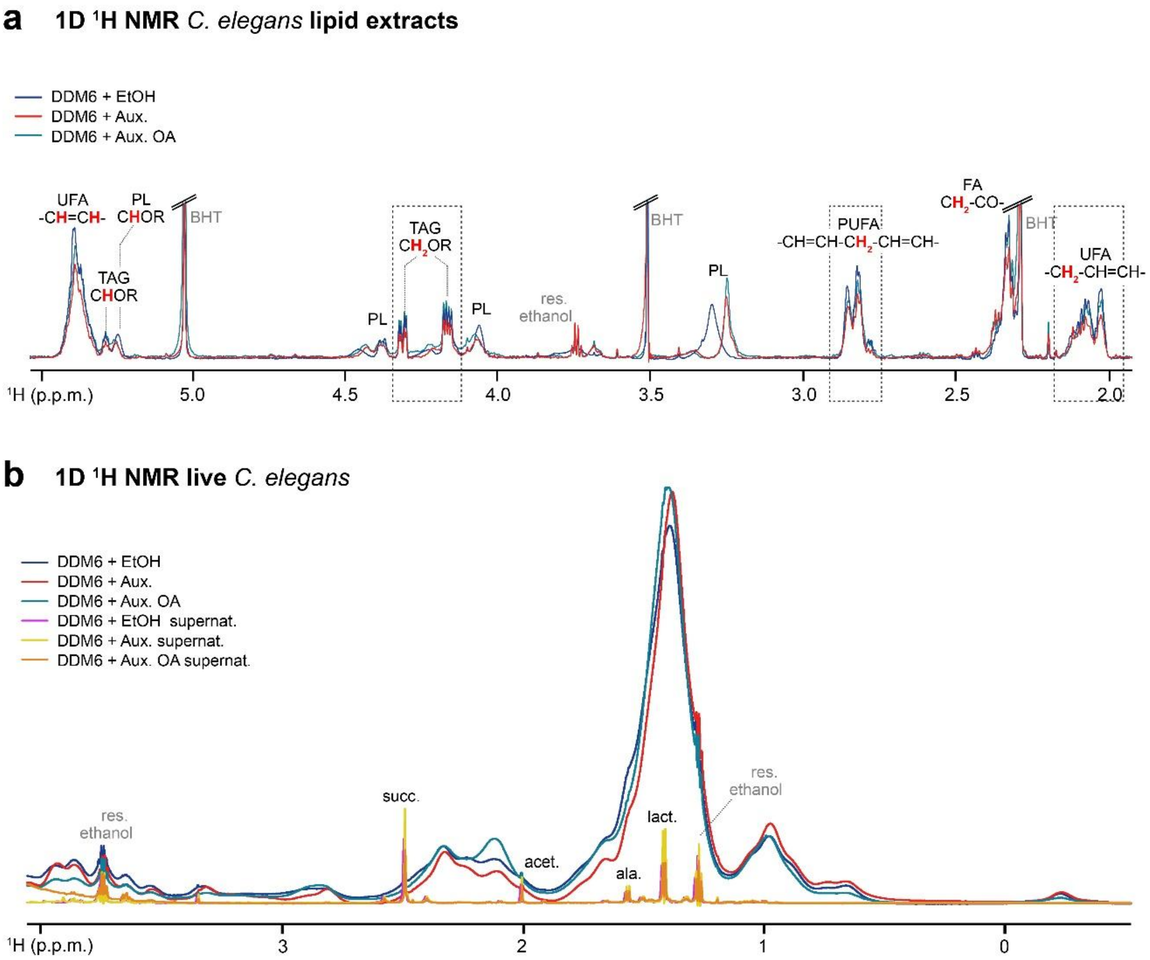

Figure S4. (a) 1D ^{1}H NMR spectra of *C. elegans* lipids extracted from DDM6 worms treated with ethanol (blue), auxin (red) and auxin and oleic acid (cyan). Dotted squares indicate the TAG, PUFA and UFA spectra regions enlarged in figure 2e. BHT, butylated hydroxytoluene: res. ethanol, residual ethanol. **(b)** 1D ^{1}H NMR spectra of ^{13}C-isotopically enriched DDM6 worms and the corresponding supernatant separated after *in vivo* acquisitions. Only a restricted set of low intensity metabolites are present in the supernatant, indicating that worms were intact during *in vivo* NMR experiments and that the lipid signals detected arise from within the animals. Excreted metabolites correspond to succinate (succ.), acetate (acet.), alanine (Ala) and lactate (lact.). In all cases DDM6 worms treated with ethanol are shown in blue, with auxin in red and with auxin and non-isotopically enriched oleic acid in cyan. The corresponding supernatants are depicted in magenta, yellow and orange, respectively.

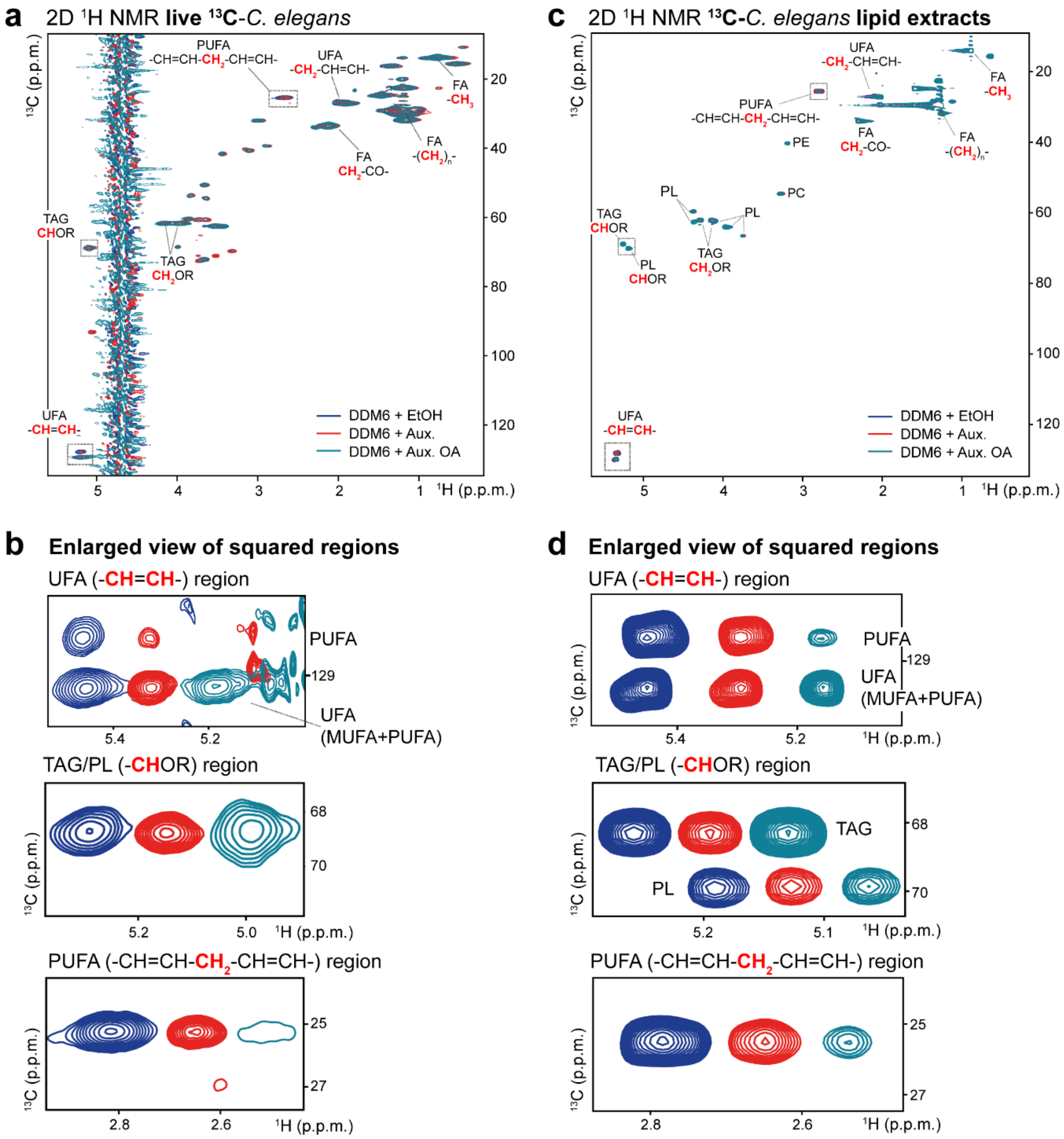

Figure S5. 2D ^{1}H-^{13}C HSQC spectra of live ^{13}C-isotopically enriched DDM6 worms **(a, b)** and lipids extracts **(c, d)**. Dotted squares indicate the TAG/PL, UFA and PUFA regions and are enlarged in panels **(b)** and **(d),** respectively. The ^{13}C traces from the UFA, TAG and PUFA signals are shown in **Figure 3e, f**. In all cases DDM6 worms were treated with ethanol (blue), auxin (red) and auxin and non-isotopically enriched oleic acid (cyan).

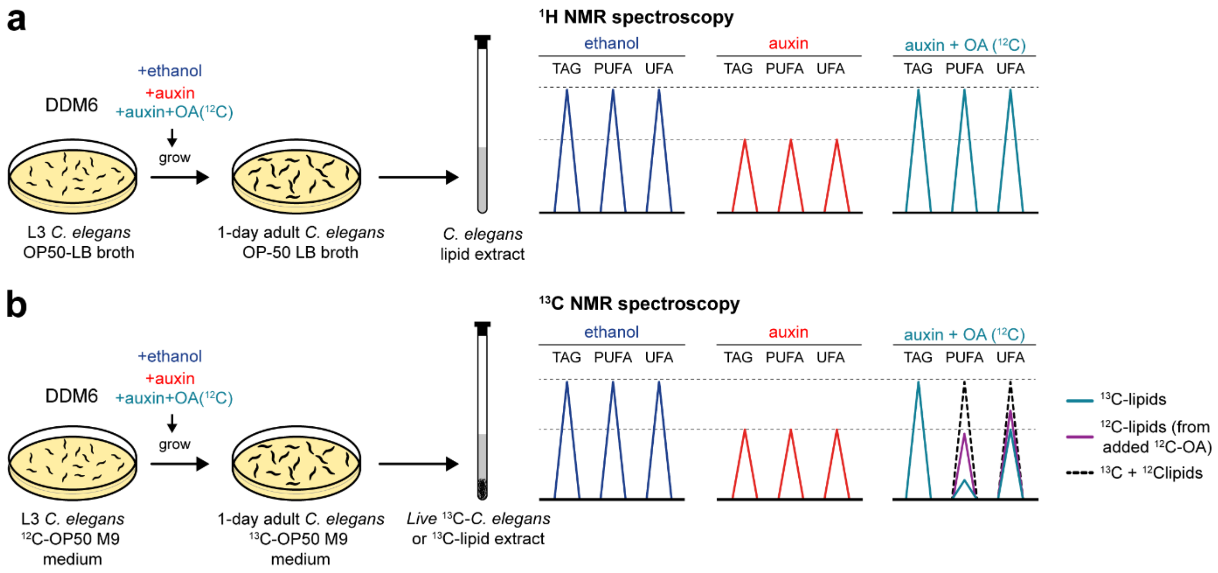

Figure S6. NMR of lipid extracts and live *C. elegans* fed with non-labeled and uniform ^{13}C-isotopically enriched OP50 bacteria. **(a)** Schematic representation of ^{1}H NMR signals of TAG, UFA and PUFA of non-enriched worms. TAG and unsaturated fatty acyl chains from *de novo* synthesis, incorporated from the bacteria (palmitoleic and cis-vaccenic acids) or from exogenous OA contain ^{1}H and are NMR visible. **(b)** Schematic representation of ^{13}C NMR signals of TAG, UFA and PUFA of ^{13}C isotopically enriched worms. In the ethanol and auxin conditions, TAG, UFA and PUFA are synthetized *de novo* or incorporated from ^{13}C enriched bacteria, thus they are NMR visible. In the auxin+OA (^{12}C), TAG glycerol groups from *de novo* synthesis contain ^{13}C and are NMR visible (light blue line). UFA from *de novo* synthesis or incorporated from the bacteria (palmitoleic and cis-vaccenic acids) contain ^{13}C and are NMR visible (light blue line) while UFA derived from exogenous OA contain ^{12}C and are NMR invisible (purple line). PUFA come from exogenous OA, contain mostly ^{12}C and are NMR invisible (purple line). The dotted black line represents the signal expected by the combination of ^{13}C and ^{12}C lipids if all of them were NMR visible.

Fatty Acid	DDM6+EtOH	DDM6+Aux	DDM6+AUX+OA	N2+EtOH
**14:0 n**	2.9 ± 0.5	2.4 ± 0.7	0.84 ± 0.05 *	2.4 ± 0.8
**16:0 n**	6.1 ± 0.6	8.3 ± 1.0	4.7 ± 2.1	5.5 ± 1.4
**18:0 n**	21.2 ± 0.6	37.4 ± 1.5 ***	22.7 ± 3.0	14.7 ± 2.5
**Total saturated**	30.2 ± 1.6	48.2 ± 3.0 **	28.35 ± 5.1	22.7 ± 4.5
**16:1 cis**	0.28 ± 0.09	0.5 ± 0.05	0.4 ± 0.07	1.6 ± 0.4 *
**18:1Δ9**	5.4 ± 1.0	1.3 ± 0.3 **	7.9 ± 1.2	3.4 ± 0.4
**18:1Δ11**	8.4 ± 2.6	8.4 ± 2.0	9.1 ± 1.1	10.4 ± 2.7
**Total MUFA**	14.1 ± 3.3	10.23 ± 2.4	17.4 ± 2.2	15.5 ± 3.6
**18:2 n6**	15.1 ± 2.4	7.4 ± 0.4 *	15.1 ± 5.7	12.7 ± 0.2
**20:3 n6**	2.2 ± 0.5	1.9 ± 0.05	3.1 ± 0.2	2.8 ± 0.9
**20:4 n6**	1.0 ± 0.08	1.3 ± 0.4	1.4 ± 0.2	1.2 ± 0.2
**20:4 n3**	4.3 ± 0.4	2.9 ± 0.05 *	5.3 ± 0.4	5.6 ± 1.1
**20:5 n3**	12.6 ± 2.0	5.8 ± 0.5 *	15.2 ± 0.7	12.9 ± 2.2
**Total PUFA**	35.2 ± 2.7	19.4 ± 0.9 **	40.0 ± 6.9	35.3 ± 3.9
**Total UFA**	49.3 ± 5.1	29.7 ± 3.1 *	57.4 ± 5.1	50.7 ± 1.9
**C15 iso**	6.4 ± 2.1	6.5 ± 1.1	3.0 ± 0.3	7.1 ± 1.9
**C17 iso**	9.9 ± 2.4	8.5 ± 0.6	5.5 ± 0.6	14.4 ± 2.5
**Total branched**	16.3 ± 4.5	14.9 ± 1.7	8.5 ± 0.6	21.6 ± 4.2
**17Δ**	2.6 ± 1.0	5.0 ± 1.8	4.6 ± 1.2	2.6 ± 2.2
**19Δ**	1.4 ± 0.2	2.1 ± 0.5	1.0 ± 0.3	2.3 ± 2.0
**Total cyclopropane**	4.0 ± 0.9	7.1 ± 1.3	5.6 ± 1.2	4.9 ± 3.5

Table S1. Data are weight percentages (Mean+SEM) of four to six independent determinations of total worm fatty acids measured by gas chromatography. For each determination L3 larvae were placed on auxin, ethanol or auxin + oleic acid supplemented NGM plates until they reached 2-day-old adult stage. 17Δ, 9,19-methylenehexadecanoic acid, 19Δ, 11,12-methyleneoctadecanoic acid. Values determined to be significantly different from DDM6+EtOH worms using an unpaired *t*-test are (*) $p < 0.05$; (**) $p < 0.01$; (***) $p < 0.001$ and (****) $p < 0.0001$.
